# Stemming the Rising Toll of People Living with Complex Care Needs

**DOI:** 10.1001/jamahealthforum.2020.0282

**Published:** 2020-04-01

**Authors:** Mary D. Naylor, Brianna Morgan, Ashley Z. Ritter

**Affiliations:** NewCourtland Center for Transitions and Health, University of Pennsylvania, Philadelphia; NewCourtland Center for Transitions and Health, University of Pennsylvania, Philadelphia; NewCourtland Center for Transitions and Health, University of Pennsylvania, Philadelphia; National Clinician Scholars Program, University of Pennsylvania, Philadelphia

Many people in our society grapple with multiple substance abuse, behavioral, and physical health problems. Clinicians regularly serve people who rely exclusively on hospitals to meet their complex needs. The health care system characterizes these individuals as superutilizers.

Widespread attention to the inadequacies of our current health care system in responding to the rapidly growing segment of society confronting similar issues has stimulated well-motivated innovations. Typically designed to provide the most vulnerable individuals in society with a range of services at arguably the most challenging time in their lives, most such programs have failed to achieve desired outcomes. From our perspective, shepherding meaningful advances in stemming the rising toll of adults living with complex health and social needs requires the simultaneous pursuit of micro-level and macro-level solutions focused on at-risk individuals and communities. Findings from rigorously studied interventions should inform the design and evaluation of future solutions. In the short-term, we need an effective and actionable path for immediate change.

For people with complex care needs, repetitive and costly interactions with the health care system are the downstream effects of persistent, unmet health and social needs. We agree with scholars who argue that addressing the needs of at-risk populations requires upstream, macro-level interventions that target poverty, food insecurity, housing instability, social isolation, and related issues. Indeed, solutions of this nature are critical drivers of social and economic change and, ultimately, the elimination of health inequities.^1^ For example, Partners for Rural Transformation, a national coalition supported by the Robert Wood Johnson Foundation, seeks to enhance economic mobility in communities experiencing sustained poverty. To date, evidence of this initiative’s success includes the creation of public and private partnerships that have secured financial investments in community organizations and policy recommendations aimed at procuring federal funding for chronically poor rural communities. However, critically needed responses such as this initiative typically require substantial, long-term investment with health benefits that may only be observable after many years or in future generations. Thus, there is a crucial need to simultaneously pursue preventative approaches at the individual level capable of achieving immediate change.

There is rigorous evidence that interventions targeting specific individuals at especially vulnerable times have positively changed the trajectories of their lives and substantially benefited society. The Nurse Family Partnership, for example, provides first-time, low income mothers with home visits by nurses throughout their pregnancies and until their children reach 2 years of age. This program, designed to improve health outcomes for mothers and children while promoting economic self-sufficiency, has improved both short-term outcomes (eg, significant reductions in preterm deliveries) and longer-term outcomes (eg, higher graduation rates among children in this program compared with similar at-risk youth),^2^ with an estimated savings to individuals and communities of $18 000 per participant after accounting for the program’s costs.^3^

Over time, both individuals and communities inevitably confront the accumulation of health challenges associated with aging that require different micro-level and macro-level solutions. Project HOME addresses the drivers of homelessness in North Philadelphia through housing investments, integrated health services, and life skills to build a community for individuals experiencing chronic physical and behavioral health conditions. This nonprofit organization has created nearly 1000 housing units since its inception in 1989 and now supports 550 jobs annually while fostering housing stability and improved health outcomes for community members.

The Transitional Care Model, an advanced practice registered nurse-coordinated, team-based intervention, seeks to interrupt the downward trajectory of chronic illness that contributes to older adults’ frequent use of costly services. In addition to assuring access to health and social services to meet immediate goals and needs, this model has demonstrated longer-term outcomes by having the same advanced practice registered nurse provide continuity of care across diverse teams and settings. In multiple studies, the Transitional Care Model has demonstrated improvements in older adults’ care experience, health, and quality of life,^4^ with estimated yearly net savings of $4500 per older adult.^5^

While scholars continue to debate the value of micro-level vs macro-level solutions, we believe strongly that both are critical to success. Thus, public and private investments in innovations designed to address the foundation of health inequities as well as health care models targeting those suffering the devastating consequences of a lifetime of health assaults should both be a priority. These investments, however, should be informed by a systematic assessment of lessons learned from studies of existing innovations, including programs that did and did not meet desired objectives.

Key ingredients inferred from available solutions suggest the importance of clearly defined target populations and early intervention as well as the value of holistic, multidimensional approaches grounded in trust, with fully engaged partners. Future studies should clearly elucidate core components of interventions and identify facilitators and barriers to their implementation. To identify effective approaches, outcome metrics that are of importance to targeted recipients and that closely align with the goals of individuals and communities, such as functional status or quality of life, are needed. The use of both qualitative and quantitative methods in evaluations is essential to maximize learning and inform broader implementation and scaling. Indeed, future investments should be accompanied by a commitment to employing a learning health system model designed to rapidly share lessons with the range of stakeholders engaged in this challenging work.

While awaiting outcomes from this path, fostering the adoption or adaptation of rigorously proven solutions represents an immediate opportunity to improve outcomes for this complex population. Capitalizing on emerging policies that foster accountable health communities or promote better integration of health and social services as well as prioritizing payment for evidence will accelerate the spread of such innovations.

People with complex health care needs deserve our best evidence-based care today. Future generations deserve investments in both micro-level and macro-level solutions that will stem the tremendous human and economic consequences of living with complex care needs and give all members of society a better chance of living healthier, higher-quality lives.
